# On habit and the mind-body problem. The view of Felix Ravaisson

**DOI:** 10.3389/fnhum.2014.00684

**Published:** 2014-09-09

**Authors:** Leandro M. Gaitán, Javier S. Castresana

**Affiliations:** ^1^Unit of Medical Education and Bioethics, University of Navarra School of MedicinePamplona, Spain; ^2^Department of Biochemistry and Genetics, University of Navarra School of SciencesPamplona, Spain

**Keywords:** habit, mind-body problem, metaphysics, anthropology, consciousness

In his book *De l'habitude* (*Of Habit*), 1838, the philosopher and archeologist Felix Ravaisson deals with the study of habit by using a broad spectrum of sources ranging from Aristotle to Butler, Leibniz, Hume, Main de Biran and Schelling, among others. The combination of these authors together with the originality of Ravaisson's own results produces a work which, though brief, has inspired some of the most important contemporary philosophers^1^. Moreover, this book has recently (2008) been translated into English for the first time, which has favored its rediscovery and redefinition in the context of current debates. Indeed, Ravaisson seems to have found in the study of habit a key point for the solution of some of the fundamental problems of philosophy such as the relationship between mind and body, nature and freedom, and nature and culture (Carlisle, 2013). Moreover, his approach is distinguished from the dominant method that from Descartes onwards has been focused on the study of consciousness rather than precisely on habit.

Ravaisson's approach currently remains as challenging as in his own time. This is because the anthropological conception of cognitive science is based on a clearly defined and tacitly assumed axiom: that human beings are essentially thinking beings, demonstrating that cartesianism is as valid today as in the days of Ravaisson. And for that reason, the traditional position of neuroscience tends to ignore the importance of habit (Noë, 2009). In this article we will examine how the study of the nature of habit applies to the mind-body problem and discuss the ontological status of habit, as well as the habit-consciousness relationship.

In his essay *La vie et l' oeuvre de Ravaisson* (1938/2009), the philosopher Henri Bergson says that the work *Of Habit*, despite having a modest title, is a treatise on the philosophy of nature, as it offers answers to key questions such as “What is nature? How to represent its inside? What does it hide under the regular succession of causes and effects? Does it cover something or is it reduced, in short, to a whole array of completely superficial movements that mechanically engage one another?” (pp. 266–267). This last question is key regarding the issue addressed here. Ravaisson, naturally reluctant to the great metaphysical constructs, will find the right tool to answer this question in such a daily occurrence as the habit. According to Bergson's interpretations, the inner experience shows that the habit is an activity that has passed, by insensible degrees, from consciousness to unconsciousness, and from voluntary to involuntary action. This seems to suggest that nature is a kind of obscured consciousness, or sleepy will^2^.

Now, why does the habit play such a crucial role in the ravaissonian conception of the mind-body or mind-matter problem? One could start by saying that the habit “is […] a disposition relative to change, which is engendered in a being by the continuity or the repetition of this very same change” (Ravaisson, 1838/2008; p. 25) and is “a general, permanent way of being” (Ravaisson, 1838/2008; p. 25). The habit, according to Ravaisson, is not possible at the inorganic level (physical, chemical, and mechanical), but it is organically possible. This is because the physical bodies are subject to external influences, i.e., to the general laws of matter, while living things have a nature that remains constant in the midst of change. For this reason, there is individuality only where there is life.

The author defines the realm of the inorganic as the “empire of Destiny,” and the organic realm as the “empire of Nature” (Ravaisson, 1838/2008; p. 31). So, the habit occurs, ontologically speaking, in nature, in the living world. And the law of habit is the development of a spontaneity that runs through the dichotomy between the “mechanical Fatality” and the “reflective Freedom,” as it is not identified with either of those. The habit is an “inclination that follows from the will” (Ravaisson, 1838/2008; p. 55), i.e., an idea—result of reflection and willingness—that gradually transforms in being, in “substantial idea” (Ravaisson, 1838/2008; p. 55) or in “thought in action” (Ravaisson, 1838/2008; p. 59). In other words, an idea that gradually naturalizes, an action that, as a result of repetition, imperceptibly moves from the understanding and the will, to nature. So Nature is the limit of habit: “In descending gradually from the clearest regions of consciousness, habit carries with it light from those regions into the depths and dark night of nature. Habit is an acquired nature, a second *nature* that has its ultimate ground in primitive nature, but which alone explains the latter to the understanding” (Ravaisson, 1838/2008; p. 59). The purely biological sphere is a sort of lower limit, while the sphere of reason and the will is the upper limit. Therefore, the habit flows from the upper limit to the lower limit, revealing a continuity underlying along the whole spectrum^3^.

Certainly, it is in connecting those limits that habit plays a more prominent role and in which is revealed as a key to search for answers to the mind-body problem. As mentioned above, the habit is an action that harmoniously unites the area of freedom, intentionality, reflection and will with our most primitive nature^4^ and includes therefore two vectors: an open *temporality* in which the future is not contained in the present, but where the present places certain regularities or patterns that anticipate what the future may include; and a *living being* whose activities may be modified by the incorporation of stereotyped behaviors (Grosz, 2013). Considering both vectors, the habit can be conceived as a complex phenomenon that is part, concomitantly, of our consciousness, and of our natural tendencies or impulses. One could argue that the habit is, then, a kind of instinct, or learned impulse that becomes standard of behavior.

But Ravaisson is cautious in speaking of habit and instinct. These functions are not identifiable because there is a difference of degree among them. Instinct is thoughtless, necessary, and perfectly spontaneous; devoid of any will and consciousness. The habit, however, has its starting point in consciousness and never completely ignores it (Malabou, 2008). However, the difference between habit and instinct can be reduced *ad infinitum* as the habit is strengthened by repeated and prolonged exercise. As pointed out by one of its most important scholars, in habit “the facility in an action gained through its repetition can become a pre-reflective desire, tendency or inclination to carry out the act […] but this inclination, in turn, can develop into the almost completely involuntary phenomena that we know as tics” (Sinclair, 2011a,b). This ravaissonian idea is deeply original and important, because it highlights an aspect that is not present in other authors (including neuroscientists and contemporary philosophers). This aspect refers to the existence of an imperceptible gradualness in the process of acquiring the habit, and therefore, to the existence of habits with different degrees of strengthening or consolidation. For example, novice driver has certain visual-motor skills that undoubtedly constitute a habit. But the level of strengthening of that habit is not comparable to the case of a rally driver.

In the novice driver, the habit is not yet sufficiently near to the lower limit. In between the extremes—the beginner level and expert level—, there are countless intermediate levels. In the beginner, reasoning and free will still have a huge role, while the habit of driving is almost instinctive in the expert driver. According to the ravaissonian thesis, there seems to be an inverse relationship between consciousness and habit: more consciousness, less habit; more habit less consciousness. However, it should be stressed that, according to the author, at no time is consciousness completely eliminated. Recently, neuroscience has verified Ravaisson's assertions: experts with very entrenched habits significantly drop their brain activation level; that is, the more established you have a habit, the brain must work less. Which implies a significant reduction in muscle activity, gain in precision and elegance, and energy savings (Noë, 2009).

It is also possible to correlate the philosophical concept of habit and brain plasticity. The presence of habits in the organic world reveals the existence of a limit for change. Without habits, lifetime would be subject to the circumstances and completely adrift. Conversely, if habits would prevent any possibility of change, life would be reduced to a mere mechanism. The concept of plasticity, understood in the terms applied to the brain, i.e., its own ability to change itself, summarizes the two conditions of habit: (a) the condition of resistance to change; and (b) the condition for flexibility and variation. In other words, the habit is a form of resistance to change gradually acquired, that shows at the same time, the ability of living beings to change. On this, Carlisle states: “…while contemporary accounts of the brain's plasticity help us to understand the processes of habit formation, philosophical reflection on habit helps us to understand the significance of plasticity”^5^ (Carlisle, 2014; p. 22). Therefore, Ravaisson's ideas about the habit and the theory of neural plasticity can be mutually reinforcing.

On the other hand, the process of acquiring the habit modifies both the mind and body, “there is, therefore, a single force, a single intelligence that is, in the life of man, the principle of all this functions and forms” (Ravaisson, 1838/2008; p. 65). According to the latter reference, the mind-body relationship would not be explained as the articulation of two substances, even two properties. Mind and body form the ends (upper and lower limits) of a *continuum*^6^ in which the habit gradually down-flows. Certainly Ravaisson admits it is not possible to apodictically prove the absolute continuity between the two limits, and therefore, the existence of one and the same principle. The *continuum*, the underlying dark unit, harmonizing principle postulated by the philosopher of Namur, is only a possibility and an assumption that cannot be verified in nature. However, this presumption is inferred from the progression of habit because “… it draws its proof from it, by the most powerful of analogies” (Ravaisson, 1838/2008; p. 65). Ravaisson's argument compels us to think the habit from outside the predominant dualistic paradigm of modernity, and offers a phenomenological and metaphysically superior explanation.

Then, the habit is not a mere accident in the world of life, but the key to their organization and their subsistence, being a structural component in it, regardless of their level of complexity or stage of development. On the other hand, considered from the social point of view, “When we contract habits from others by sharing spaces, practices, routines and rhythms, and a language, communication and interaction become easier and less effortful, and communal life becomes more harmonious” (Carlisle, 2013). The habit, whatever the angle from which it is considered, is a unifying element that reveals the existence of continuities in the human being individually or collectively understood. Where there is habit, there is order and connection. Considering all this, it is not absurd to claim that the habit is the clearest expression of the *continuum*. This seems to be the ontological value of the habit in Ravaisson's work.

But this is not all. It should be added that the habit does not mechanize or reduce consciousness to unconsciousness or to mere automatism, but “it brings about a new kind of consciousness, one not aware of itself but prone to act, that is activated by the possibility of its acting, that knows but cannot know that it knows” (Grosz, 2013). The ravaissonian conception of consciousness differs substantially from the cartesian conception that identifies it with reflective thought, will and therefore with knowledge. The ravaissonian consciousness has degrees (like the habit); in fact, in some of them it does not know but it acts, and acting produces effects (actions and feelings); that consciousness is always near instinct, and in its daily application through habit it opens to the possibility of creation, transformation, and learning. Thus seen, the habit, far from being mere mechanical automation, is possibility of innovation through the acquisition of new traits and skills, and openness to the future.

The author shows the habit manifests the inhabitation of freedom and intelligence in the body (Carlisle, 2013). Indeed, the process of acquiring the habit involves a shift from free reflection to the primitive nature in order to obtain that second nature (to which we refer above), but this in turn serves as a platform for further actions of free reflection. Put succinctly, the habit is the condition of possibility of conscious actions. For example, if a musician composes a song, the realization of this purpose involves the previous acquisition of physical and intellectual habits such as management of musical instruments and of singing techniques, mastery of musical notation and music theory, etc. The most original manifestations of intelligence and freedom are the result of habit.

So, the habit operates in two directions fulfilling a sort of recursive function within the mind-body *continuum*: downwards, ranging from consciousness to nature (in the process of acquisition); and upwards, ranging from nature to consciousness (once it has taken hold). This double movement attributed by Ravaisson to habit, shows an original anthropological conception, refractory of any dualism or reductionism. Indeed, according to the philosopher, humanity is not confined to the *res cogitans*, or mere *brainhood* (as postulated in the mainstream of current neuroscience). The human being is an embodied subjectivity, is a *self*, the most genuine form of unity.

Thus, the study on habit done by Ravaisson offers a phenomenologic-metaphysical answer to the so-called *hard problem* of philosophy of mind; an answer long forgotten and hard to locate in the complex map of current theories, which can still provide interesting clues not only to philosophy but also to the current neuroscience of habit.

## Conflict of interest statement

The authors declare that the research was conducted in the absence of any commercial or financial relationships that could be construed as a potential conflict of interest.

## Footnotes

^1^Maurice Merleau-Ponty and Paul Ricoeur in phenomenology, Henri Bergson and Gilles Deleuze in vitalism, and William James and John Dewey in american pragmatism.

^2^Bergson finds in Ravaisson the bases of his theory of *élan vital*, and of nature as obscured consciousness.

^3^Ravaisson refers primarily to motor habits.

^4^The ravaissonian thesis unifying the ideal and the reality by habit, reflects the influence of Schelling.

^5^For a neuroscientific approach to processes of habit formation, see Graybiel (2008).

^6^Ravaisson inherited the notion of *continuum* from Leibniz.
